# Inequalities in education and national income are associated with poorer diet: Pooled analysis of individual participant data across 12 European countries

**DOI:** 10.1371/journal.pone.0232447

**Published:** 2020-05-07

**Authors:** H. L. Rippin, J. Hutchinson, D. C. Greenwood, J. Jewell, J. J. Breda, A. Martin, D. M. Rippin, K. Schindler, P. Rust, S. Fagt, J. Matthiessen, E. Nurk, K. Nelis, M. Kukk, H. Tapanainen, L. Valsta, T. Heuer, E. Sarkadi-Nagy, M. Bakacs, S. Tazhibayev, T. Sharmanov, I. Spiroski, M. Beukers, C. van Rossum, M. Ocke, A. K. Lindroos, Eva Warensjö Lemming, J. E. Cade

**Affiliations:** 1 Nutritional Epidemiology Group (NEG), School of Food Science and Nutrition, University of Leeds, Leeds, England, United Kingdom; 2 Clinical and Population Science Department, Institute of Cardiovascular and Metabolic Medicine (LICAMM), University of Leeds, Leeds, England, United Kingdom; 3 Division of Noncommunicable Diseases and Promoting Health through the Life-Course, World Health Organization Regional Office for Europe, UN City, Copenhagen, Denmark; 4 Academic Unit of Health Economics, Leeds Institute of Health Sciences, University of Leeds, Leeds, England, United Kingdom; 5 Department of Environment and Geography, University of York, Wentworth Way, Heslington, York, England, United Kingdom; 6 Department of Nutritional Sciences, University of Vienna, Vienna, Austria; 7 National Food Institute, Kemitorvet, Lyngby, Denmark; 8 Department of Nutrition Research, National Institute for Health Development, Tallinn, Estonia; 9 Department of Nutrition, Institute of Basic Medical Sciences, University of Oslo, Oslo, Norway; 10 Public Health Promotion Unit, Finnish Institute for Health and Welfare, Helsinki, Finland; 11 Department of Nutritional Behaviour, Max Rubner-Institut, Federal Research Institute of Nutrition and Food, Karlsruhe, Germany; 12 National Institute of Pharmacy and Nutrition; Budapest, Hungary; 13 Kazakh Academy of Nutrition, Almaty, Republic of Kazakhstan; 14 Institute of Public Health, Skopje, North Macedonia; 15 National Institute for Public Health and the Environment, Bilthoven, The Netherlands; 16 Livsmedelsverket Swedish National Food Agency, Uppsala, Sweden; Institute of Economic Growth, INDIA

## Abstract

**Background:**

Malnutrition linked to noncommunicable diseases presents major health problems across Europe. The World Health Organisation encourages countries to conduct national dietary surveys to obtain data to inform public health policies designed to prevent noncommunicable diseases.

**Methods:**

Data on 27334 participants aged 19-64y were harmonised and pooled across national dietary survey datasets from 12 countries across the WHO European Region. Weighted mean nutrient intakes were age-standardised using the Eurostat 2013 European Standard Population. Associations between country-level Gross Domestic Product (GDP) and key nutrients and nutrient densities were investigated using linear regression. The potential mitigating influence of participant-level educational status was explored.

**Findings:**

Higher GDP was positively associated with total sugar intake (5·0% energy for each 10% increase in GDP, 95% CI 0·6, 9·3). Scandinavian countries had the highest vitamin D intakes. Participants with higher educational status had better nutritional intakes, particularly within lower GDP countries. A 10% higher GDP was associated with lower total fat intakes (-0·2% energy, 95% CI -0·3, -0·1) and higher daily total folate intakes (14μg, 95% CI 12, 16) in higher educated individuals.

**Interpretation:**

Lower income countries and lower education groups had poorer diet, particularly for micronutrients. We demonstrate for the first time that higher educational status appeared to have a mitigating effect on poorer diet in lower income countries. It illustrates the feasibility and value of harmonising national dietary survey data to inform European policy regarding access to healthy diets, particularly in disadvantaged groups. It specifically highlights the need for strong policies supporting nutritional intakes, prioritising lower education groups and lower income countries.

## Introduction

Malnutrition in the form of both nutrient deficiencies and over-nutrition related non-communicable diseases (NCDs) like overweight, obesity and cardiovascular disease (CVD) has been documented as reaching epidemic proportions on an international scale. Global obesity tripled between 1975–2016 [1]. In 2018, 59% of adults in the WHO European Region were overweight or obese and NCDs including diabetes, hypertension, cardiovascular diseases, cancer and chronic respiratory diseases are the leading cause of death, disease and disability in the region [2]. In Europe 45% of deaths are attributable to CVD, with diet being the primary behavioural risk factor [3]. There is evidence that iron, calcium, vitamin D, folate and iodine are inadequately consumed in European children [4] and adults [5]. This is concerning, as deficiencies in such nutrients can lead to increase in conditions such as iron-deficiency anaemia, rickets and neural tube defects in babies [6, 7, 8]. The World Health Organisation (WHO) has developed nutrient intake guidelines underpinned by the e-library of Evidence for Nutrition Actions (eLENA), which can form the basis of monitoring programmes, and assist governments in formulating policy to improve diet quality.

The WHO European Food and Nutrition Action Plan aims to reduce the impact of malnutrition in the WHO European region, starting with more effective monitoring through national dietary surveys [9]. Monitoring enables the identification of trends, dietary inadequacies and inequalities, which can help inform and evaluate more targeted policies to improve population health across the WHO European Region. Current monitoring within the region is incomplete, with particularly sparse coverage of Central and Eastern Europe [10]. This is concerning, as nutrition policies in these countries may therefore lack an appropriate evidence base.

WHO recommended nutrient intakes (RNIs) of both macro and micronutrients are not widely achieved [5]. Despite evidence that higher socioeconomic status is associated with better diet quality globally [11, 12], few WHO European Member States report intakes by socioeconomic group [5]; this information would facilitate monitoring of potential health inequalities [9].

This research therefore aims to harmonise national individual level dietary survey data from across the WHO European Region, exploring geographical variations in key nutrient intakes, standardised using the European Standard Population (ESP). It also aims to investigate both between and within-country socioeconomic inequalities through measures of country-level Gross Domestic Product (GDP) and individual-level education.

## Methods

### Harmonisation and pooling of national surveys

National dietary surveys were identified from published summary reports, as previously reported in detail [10]. Briefly, authors of national dietary surveys from WHO European Member States were identified using two main dietary survey reports [13, 14], country responses to WHO questionnaires and further general internet searches. Contacts identified were asked to provide information on nationally representative dietary surveys conducted at an individual level since 1990. For countries where contacts could not be established, a systematic database search was performed across Web of Science, Medline and Scopus for nationally representative dietary surveys of adults and children aged over 2 years that collected data at an individual level from 1990 to June 2016. Surveys could be published or unpublished and only those based on whole diets rather than specific food groups were included. Further details of the screening process and the surveys found are presented in Rippin et al. [10].

For countries where surveys were available, individual-level nutrient intake and demographic data were requested between October 2016 and April 2018. Survey data was obtained from 12 datasets across 12 countries (Austria; Denmark; Estonia; Finland; France; Germany; Hungary; Kazakhstan; Macedonia; the Netherlands; Sweden; the UK). See S1 Appendix for details of surveys obtained. Data collection methods for the national diet surveys used included 24hr recall, consecutive and non-consecutive diaries and diet history. Data collection spanned the years 2005–2016 and sample sizes ranged from 351–10,090. However, all surveys were nationally representative.

The 12 datasets were separately cleaned, translated where necessary using Google Translate and native speaking contacts within each country, and converted to .dta format to enable statistical analysis. Key variables were re-formatted and aligned to enable analysis across datasets: unique id, weighting factor, age and sex were given common names; energy, total fat, trans fatty acid (TFA), total sugar, iron, total folate and vitamin D intakes were given common names and units of measure; and educational attainment was given a common name with aligned categories.

Macronutrients were expressed as % energy (%E), to reduce variation caused by differences in methodology or reporting [15]. Micronutrient intakes are from food only, excluding supplements. As differing numbers of dietary assessment days were collected, a mean value per individual was calculated. Children and the elderly were not included in all surveys sampled, and were therefore excluded to focus on adults aged 19-64y. Countries were grouped into three European regions: Central and Eastern, Northern, and Western.

Education level was used as an individual-level indicator of socio-economic status, as this was the only indicator present in all the diet surveys included. It could also be harmonised across all surveys to create a grouped educational attainment variable that allowed greater cross-country comparisons. This was harmonised across surveys, grouping into lower, intermediate and higher educational levels. Lower education comprised any education below secondary school level, intermediate included secondary school, college and vocational equivalents, and higher education incorporated any education beyond secondary school or college level. In the survey dataset provided by Finland, education was categorised by the total number of education years into sex and birth year-specific tertiles, to adjust for the number of years in education rather than educational attainment. This differs from other countries, but still allowed comparable educational groups to be created.

All analyses used sampling weights based on the inverse of the probability that the participant was sampled. Nine countries included a weighting variable within the dataset. Weightings were created for the three remaining countries (Kazakhstan, Macedonia, Sweden) using national population figures by age group for the latest year in which data collection took place [16]. Age-standardised mean nutrient intakes were produced using the Eurostat 2013 European Standard Population (ESP) to facilitate comparisons between countries with different population structures [17]. ESP proportions for the relevant age groups covering ages 19-64y were multiplied by the national population figures for those age groups. Population figures were taken from the latest year of data collection for the national dietary survey in question [16]. In all regression analyses using individual-level data, we used Taylor series linearization to correct the standard errors of the estimates for the sampling designs, with both inverse probability weights to weight the sample back to the population from which the sample was drawn, and stratification by survey to allow for clustering within surveys.

### Statistical analyses

Mean age-standardised daily intakes were estimated for the whole country and also by individual-level educational group and sex for each country for nutrients of concern identified *a priori*: energy (kcal/day); total fat (%E); trans fatty acid (TFA) (%E); total sugar (%E); iron (mg/day); total folate (μg/day); vitamin D (μg/day). Where total sugar intake was not reported as such (Germany, the Netherlands, Sweden), it was derived from monosaccharides plus disaccharides. To minimise risk of selection bias and because not all datasets included the variables necessary to identify and exclude under-reporters, no individuals were excluded for presumed under-reporting [18]. Two-sided p-values were used throughout and statistical significance was set at p<0·05.

Country-level age-standardised mean nutrient intakes were plotted against *per capita* GDP ($) in 2016[19, 20]. GDP was used as an indicator of socio-economic status to provide a national income perspective; individual income was not available or comparable across all surveys. Associations between nutrient intakes and GDP were estimated using linear regression. Age-standardised analyses were repeated by educational group, in men and women separately, for each country. All individual-level regression models were weighted by the inverse probability of sampling and stratified by country. The extent to which the associations between nutrient intakes and GDP differed by the participants’ educational level was estimated using linear regression, adjusted for age and sex. Similarly, the extent to which the associations between individuals’ nutrient intakes and education differed between men and women was also assessed formally by inclusion of education by sex interaction terms, adjusting for age and GDP.

GDP was log-transformed in all analyses because a non-linear relationship was predicted with diminishing marginal returns, with estimates subsequently back-transformed and presented for a 10% increase in GDP. All analyses were conducted using Stata version 15 [21].

## Results

### Harmonisation and pooling of national surveys

Datasets were obtained for 27334 participants from 12 countries within the WHO European Region: three Northern European countries (Denmark, Finland, Sweden); five Western European countries (Austria, France, Germany, Netherlands, UK) and four Central and Eastern European countries (Estonia, Hungary, Kazakhstan, Macedonia). The survey collection years spanned 2005–2016. Either 24hr recall, diet history interview or diary methods were used; see S1 Appendix for survey details. All 12 national surveys provided data on the nutrients selected for analysis, except for TFAs (not reported by Austria, France, Germany, Hungary, Sweden) and vitamin D (not reported by Austria). There were 2027 (7%) participants in the lower, 16980 (62%) in the intermediate and 8327 (30%) in the higher education groups.

### Total energy and macronutrient intakes

#### Country-level analysis

**Fig 1:**
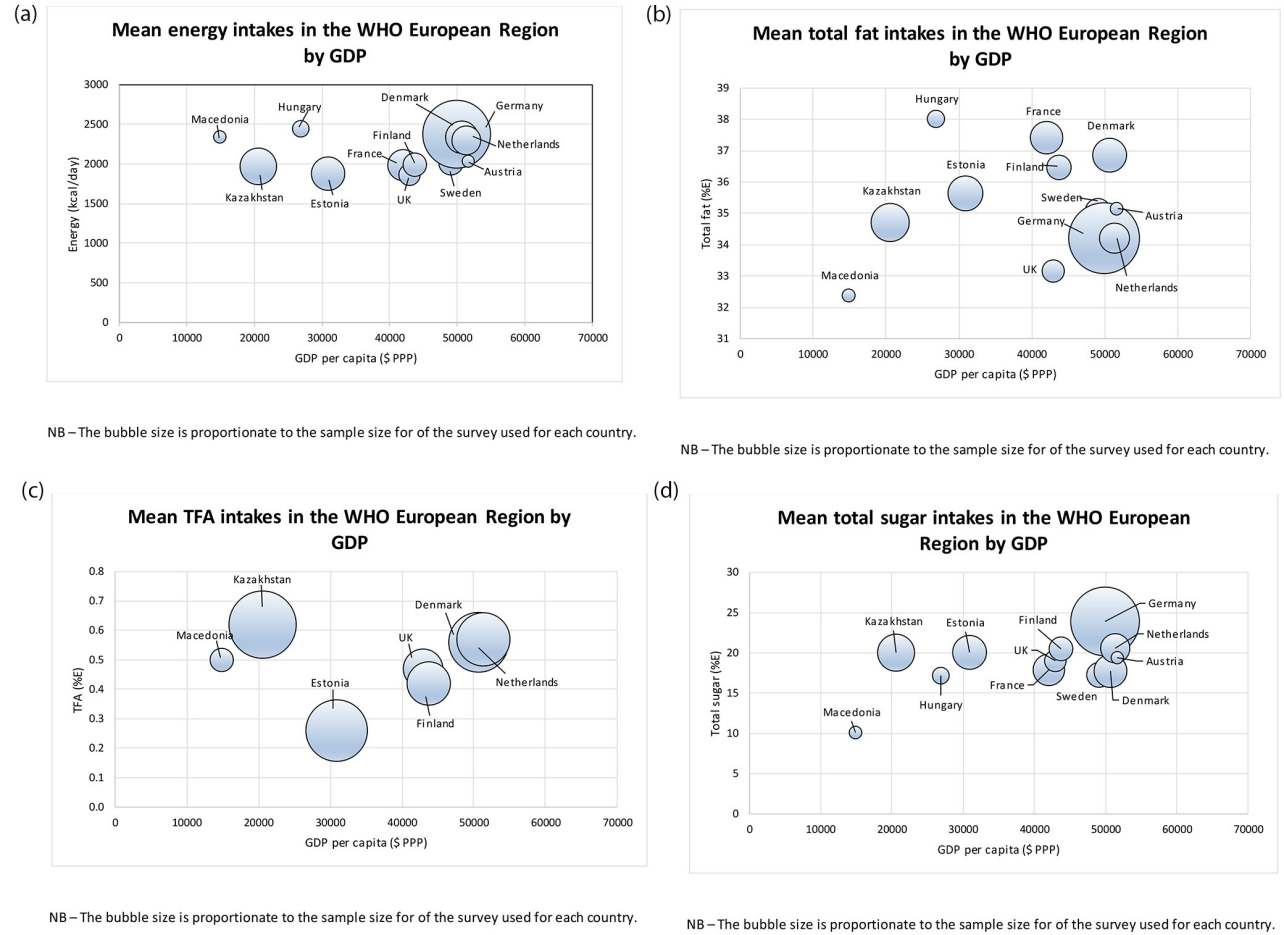
**a**–Age-standardised mean energy intakes for WHO European Member States, by GDP. **b**–Age-standardised mean % of energy from fat for WHO European Member States, by GDP. **c**–Age-standardised mean % of energy from TFAs for WHO European Member States, by GDP. **d**–Age-standardised mean % of energy from total sugar for WHO European Member States, by GDP.

**Fig 2:**
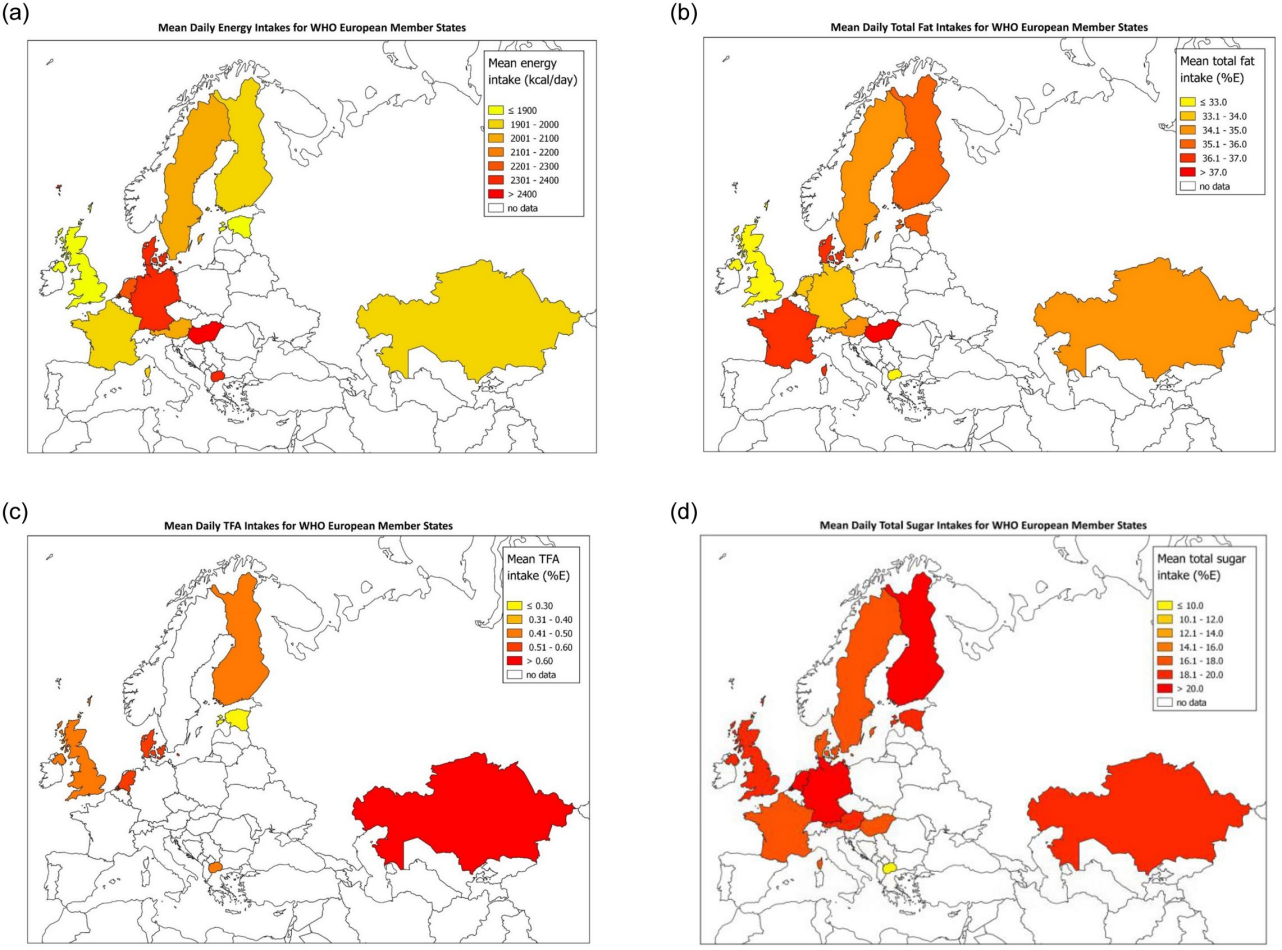
**a**–Age-standardised mean energy intakes for WHO European Member States. **b**–Age-standardised mean % of energy from fat for WHO European Member States.**c**–Age-standardised mean % of energy from TFAs for WHO European Member States. **d**–Age-standardised mean % of energy from total sugar for WHO European Member States.

Age-standardised mean daily energy and macronutrient intakes are presented in Fig 1a–1d and S2 Appendix. Between-country comparisons found that mean country energy and macronutrient intakes did not vary by national income other than in total sugar (Fig 1a–1d, S3 Appendix). Each 10% increase in GDP was associated with a mean country total sugar intake increase of 5% energy (95% CI 0·6, 9·3). Geographical variations in energy and macronutrient intakes across Europe are shown in Fig 2a–2d.

Hungarian men had the highest age-standardised mean daily energy intake (2800kcal, 95% CI 2676, 2923) and % of energy from fat (39%E, 95%CI 38, 39), and UK men the lowest (2103kcal, 95% CI 2030, 2176); (33%E, 95%CI 32, 33). Geographic variation in energy intake was similar for women, although intakes were lower and differences between countries less pronounced (S2 Appendix). In women, fat intakes were highest in France (39%E, 95%CI 38, 39) and lowest in Macedonia (32%E, 95%CI 31, 33). All countries that reported TFAs (Denmark, Estonia, Finland, Kazakhstan, Macedonia, the Netherlands, UK) had mean daily intakes below the WHO recommended <1%E. Kazakhstan had the highest intakes, with 0·63%E (95% CI 0·58, 0·68) and 0·61%E (0·56, 0·65) for men and women respectively. Estonia had the lowest intakes in both men and women, with 0·25%E (95% CI 0·24, 0·27) and 0·27%E (95% CI 0·26, 0·28) respectively (S2 Appendix). Germany had the highest male (22%E 95%CI 22, 22) and female (26%E 95% CI 26, 26) daily % of energy from total sugar. Macedonia had the lowest male and female intakes at 9%E (95% CI 7, 10) and 11%E (95% CI 10, 12) respectively (S2 Appendix). There were no apparent geographic patterns in macronutrient intakes on a European regional level.

#### Participant-level analysis

**Fig 3:**
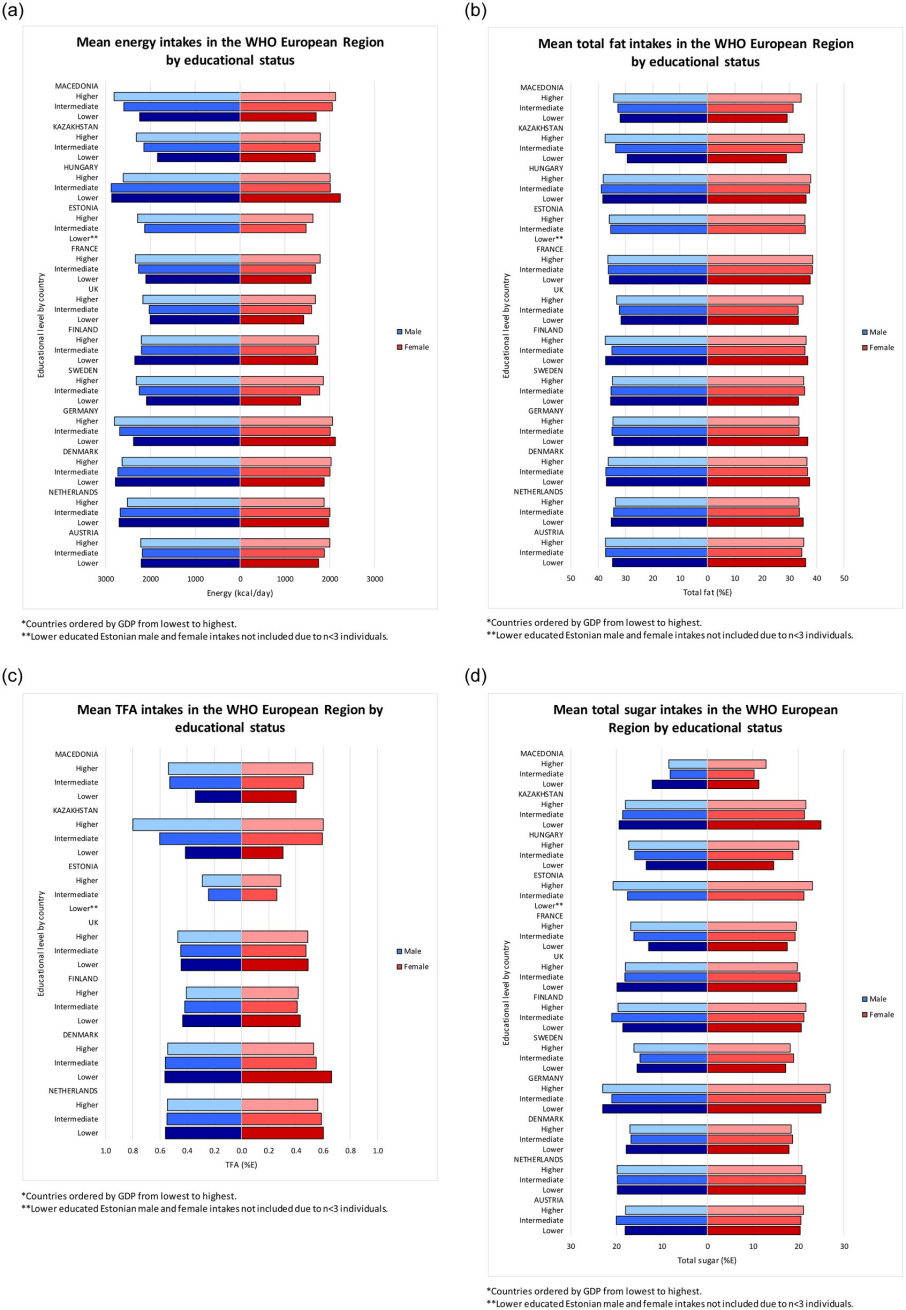
**a**–Age-standardised mean energy intakes for WHO European Member States, split by sex and education*. **b**–Age-standardised mean % of energy from fat for WHO European Member States, split by sex and education*. **c**–Age-standardised mean % of energy from TFAs for WHO European Member States, split by sex and education*. **d**–Age-standardised mean % of energy from total sugar for WHO European Member States, split by sex and education*.

Lower educational levels were associated with a lower mean energy intake in both men and women. However, this was more pronounced in lower GDP countries (Fig 3a, S4 and S5 Appendices). In lower GDP countries having a higher education level was associated with having a higher mean total fat and TFA intake, but in higher GDP countries higher educational levels were associated with lower mean fat intake. This pattern was most prominent in women (Fig 3b and 3c, S5 Appendix). The direction of associations between education and total sugar intakes varied more than in energy and fats (Fig 3d, S4 and S5 Appendices).

### Micronutrient intakes

#### Country-level analysis

**Fig 4:**
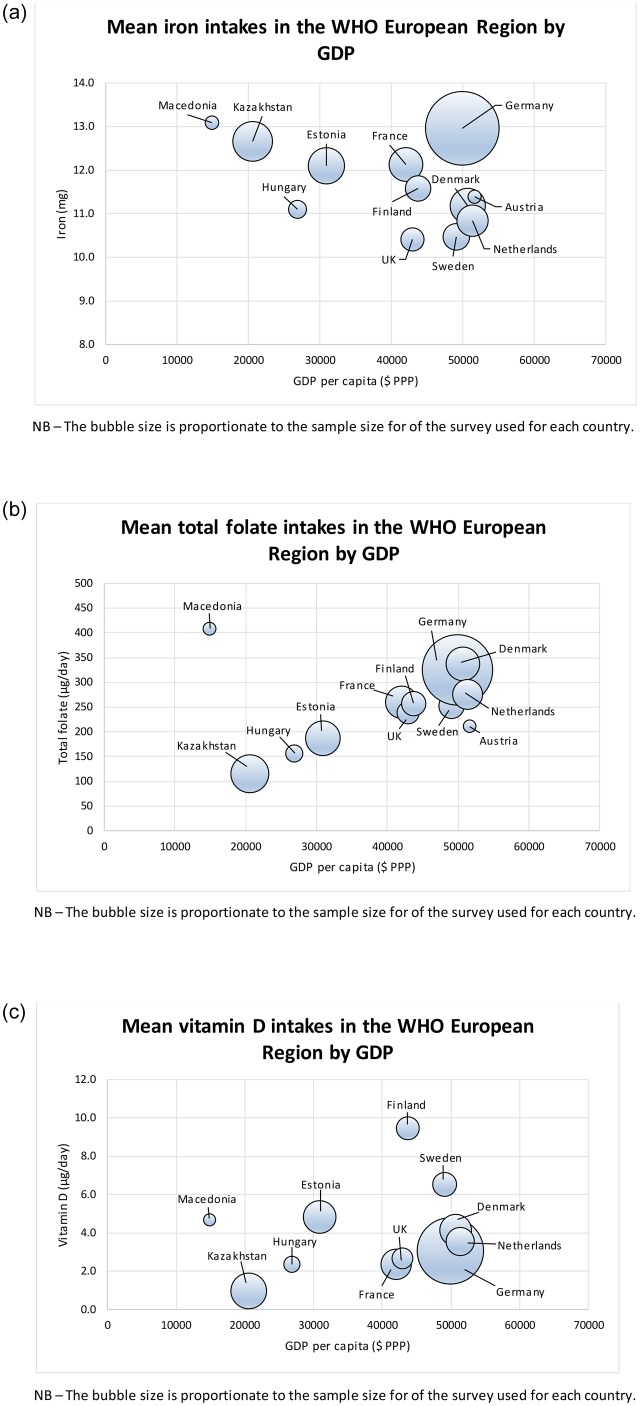
**a**–Age-standardised mean iron intakes for WHO European Member States, by GDP. **b**–Age-standardised mean total folate intakes for WHO European Member States, by GDP. **c**–Age-standardised mean vitamin D intakes for WHO European Member States, by GDP.

**Fig 5:**
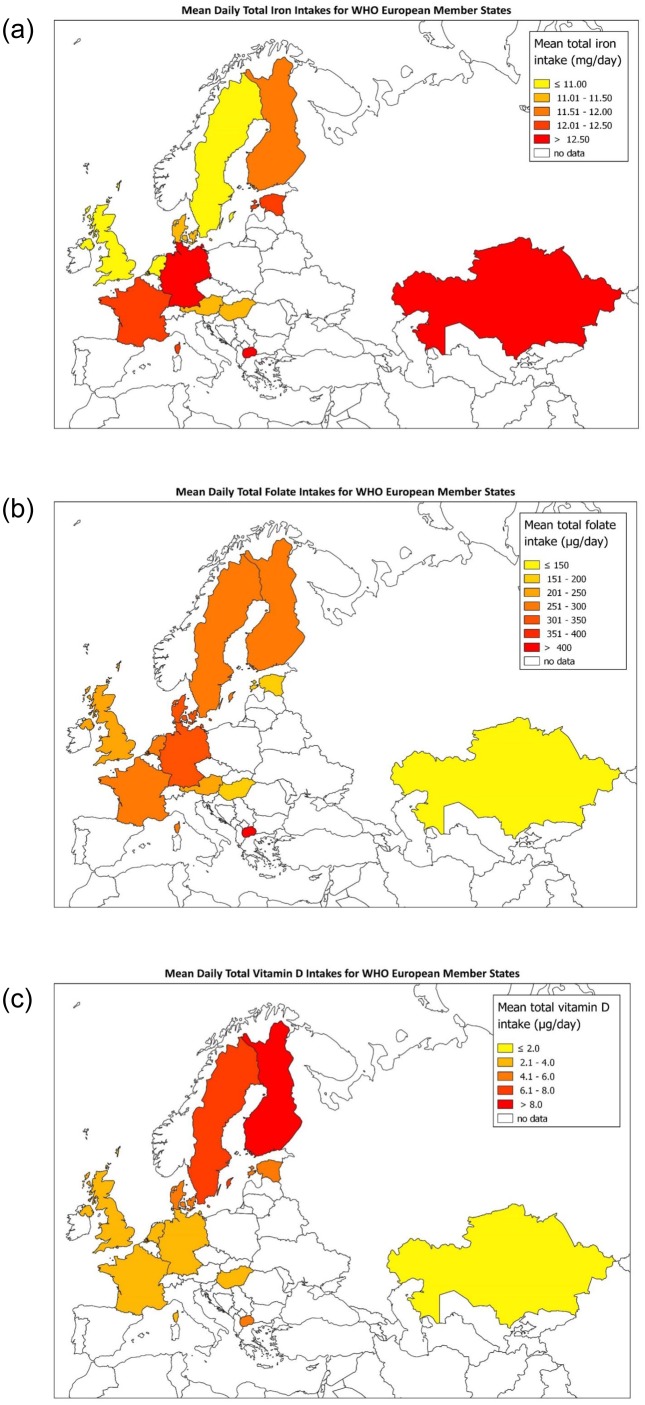
**a**–Age-standardised mean iron intakes for WHO European Member States. **b**–Age-standardised mean total folate intakes for WHO European Member States. **c**–Age-standardised mean vitamin D intakes for WHO European Member States.

Age-standardised mean micronutrient intakes are presented in Fig 4a–4c and S2 Appendix. Geographical variations in micronutrient intakes across Europe are shown in Fig 5a–5c. Mean daily iron intakes were highest in Macedonia for men and women, at 14·8mg/day (95% CI 13·8, 15·9) and 11·7mg/day (95% CI 10·9, 12·5) respectively. Sweden had the lowest male intakes (11·5mg/day, 95% CI 11·2, 11·9) and the UK had the lowest female intakes (9·2mg/day, 95% CI 8·9, 9·6). Mean daily total folate intakes were highest in Macedonian men (462μg/day, 95% CI 394, 530) and women (364μg/day, 95% CI 306, 422) and lowest in Kazakh men (124μg/day, 95% CI 121, 128) and women (107μg/day, 95% CI 104, 110). Finland had the highest mean daily vitamin D intakes in men (10·7μg/day, 95% CI 9·9, 11·4) and women (8·2μg/day, 95% CI 7·8, 8·7); Kazakhstan had the lowest, at 1·1μg/day (95% CI 1·0, 1·2) and 0·8μg/day (95% CI 0·8, 0·9) for men and women respectively (S2 Appendix). There was no evidence of associations between GDP and intakes of the included micronutrients (Fig 4a–4c, S3 Appendix). At a European regional level, the vitamin D intake range was lowest in Northern European countries, which also had the two highest intakes (Fig 5c).

#### Participant-level analysis

**Fig 6:**
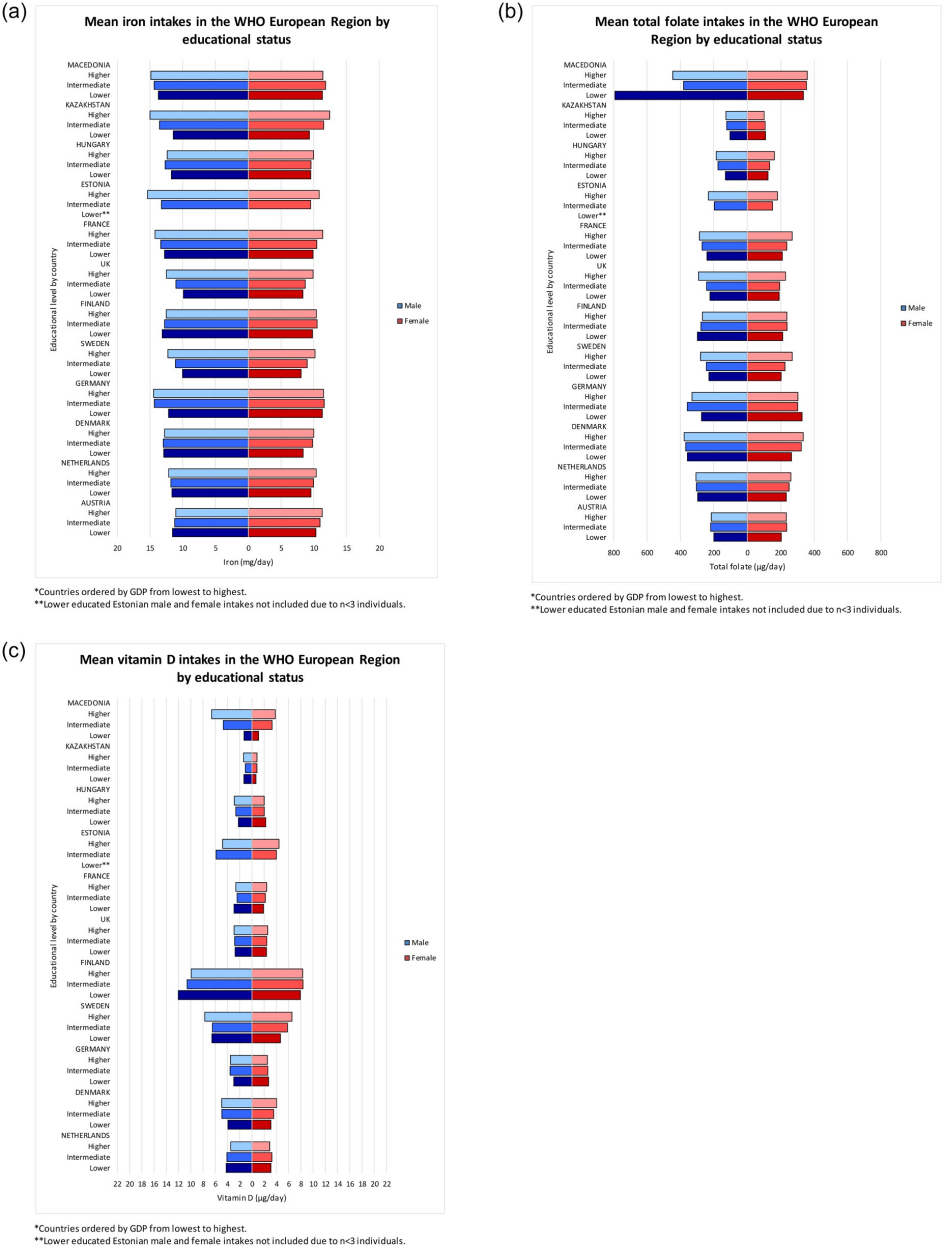
**a**–Age-standardised mean iron intakes for WHO European Member States, split by sex and education*. **b**–Age-standardised mean total folate intakes for WHO European Member States, split by sex and education*. **c**–Age-standardised mean vitamin D intakes for WHO European Member States, split by sex and education*.

Within-country comparisons found that, with few exceptions, less education was significantly associated with lower iron, total folate and vitamin D intakes in both sexes (Fig 6a–6c, S4 and S5 Appendices).

### Multiple regression analyses

After adjustment for age and sex in multiple regression models, lower GDP continued to be associated with lower individual energy intakes and lower individual vitamin intakes (Table 1). However, associations between lower GDP and both excess macronutrient and poorer micronutrient intakes were attenuated or reversed in individuals with higher educational levels. A 10% higher GDP was associated with lower total fat (-0·2%E, 95% CI -0·3, -0·1) and TFA (-0·01%E, 95% CI -0·01, -0·01) in higher education groups, whilst the associations were in the opposite direction for individuals with lower education, where higher GDP was associated with higher individual intakes (Table 1).

**Table 1:** Association between nutrient intake and GDP, by educational status, adjusted for age and sex.

	Lower Education			Intermediate Education			Higher Education			P-value**
	Slope*	95% CI		Slope*	95% CI		Slope*	95% CI		
Energy (kcal)	24	5	42	34	30	37	28	21	35	0·2
Total fat (%E)	0·2	-0·02	0·4	0·05	0·004	0·09	-0·2	-0·3	-0·1	<0·001
TFA (%E)	0·02	0·01	0·04	-0·004	-0·01	0·00001	-0·01	-0·01	-0·001	<0·001
Total sugar (%E)	0·4	0·2	0·6	0·4	0·3	0·4	0·4	0·3	0·5	0·9
Iron (mg)	-0·03	-0·16	0·09	-0·004	-0·03	0·02	-0·10	-0·16	-0·05	0·004
Total folate (μg)	2	-11	15	18	18	19	14	12	16	<0·001
Vitamin D (μg)	0·10	0·03	0·17	0·17	0·15	0·19	0·10	0·03	0·16	0·04

*Slope represents the change in nutrient intake (per unit specified) for each 10% increase in GDP.

** P-value represents the difference between subgroups defined by educational status in estimated association between GDP and nutrient intake.

Higher GDP was associated with higher daily total folate intake and vitamin D intakes for all education levels, but was most pronounced in the intermediate education group (18μg (95% CI 18, 19) for total folate; 0·17μg (95% CI 0·15, 0·19) for vitamin D). For iron, higher GDP was associated with lower intakes in all education groups, but particularly in the higher educational group (-0·1mg, 95% CI -0·16, -0·05) (Table 1). Higher GDP was associated with higher individual intakes of total sugars, but there was no evidence that this differed by individual education status (p = 0.9) (Table 1).

There was no evidence that men and women had different associations between education and nutrient intake, other than total sugar (p<0.001) and vitamin D (p = 0·004).

There was some evidence that lower education was more strongly associated with higher vitamin D intakes in men (1.4μg, 95% CI 1.0, 1.9) than in women (0.8μg, 95% CI 0.6, 1.1) (p = 0.004) (see S6 Appendix). Lower education was associated with lower daily total sugar intakes, but more so in women (-3·0%E, 95% CI -3·5, -2·4) than in men (-1·1%E, 95% CI -1·8, -0·5) (p<0.001) (see S6 Appendix). There was no evidence of other effect modification (subgroup effects) by sex (see S6 Appendix).

## Discussion

This paper presents key nutrient intakes of particular concern [9] within harmonised individual-level national survey data, pooled across 12 WHO European Member States. Each survey is sampled and weighted to provide representative data from that country, with the pooled data contributing substantial insight into dietary sufficiency across the region. Potential socioeconomic inequalities were assessed using country-level GDP and individual-level educational attainment.

Higher GDP countries had higher mean total sugar intakes. However, most countries had high energy and macronutrient intakes above WHO recommended levels; lower GDP countries may therefore face future elevated levels of obesity-related NCDs, as currently observed in higher income countries. This is already occurring [11], making government-level policy interventions imperative to prevent the situation worsening. The importance of policy is demonstrated by TFA intakes; although below the WHO <1%E recommendation, Kazakhstan had the highest mean % energy from TFAs, and until 2018, had no TFA-reduction strategy [22].

Central and Eastern European countries had lower total folate intakes, except for Macedonia. This could be explained by high national production of fruit and vegetables in Macedonia, which in turn may translate to individual diets. Northern European countries had higher vitamin D intakes, though less so in Denmark. Potential explanations include Scandinavian dietary customs such as greater oily fish consumption, which is a source of vitamin D [23]. Finland and Sweden also have extensive fortification programmes, which could explain the slightly lower Danish intakes, as Denmark does not have as strong a fortification programme [24].

Socioeconomic inequalities across WHO Europe were evident on an individual level, as men and women with less education generally had lower intakes of nutrients encouraged as part of a healthy diet, particularly iron and total folate. In some countries, lower education groups had higher intakes of energy and macronutrients, particularly total sugar. This extends into Europe evidence of a positive association between socioeconomic status and diet quality seen in the US [25, 26]. The underlying mechanism may involve diet costs; lower educated individuals may have lower paid occupations [27], resulting in less income for the range of foods needed for a balanced, healthy diet. This is compounded by lower health literacy and reduced ability to apply nutrition knowledge within budgetary constraints [28].

Greater educational attainment at individual level appeared to have a mitigating impact against the effects of low GDP for most nutrients. Although the overall effect size for individual nutrients was small, with a combined total population of almost 300 million across the 12 countries studied [16], small shifts could have an important impact on public health. National income and diet quality appear to be linked, and education could protect against some of the negative effects of poor nutrition on population health and productivity. Lower education may result in poor quality diets, and the accompanying adverse health consequences could negatively affect GDP, as less healthy individuals are less economically productive [29]. However for iron, lower intakes with higher GDP was most pronounced in the higher education groups. This may be because higher education groups in the higher income countries adopt a more plant-based, and therefore lower iron, diet [30].

These findings illustrate the importance of policy development to address the public health implications of the effect of GDP and education on nutritional intakes. The early recognition of nutrition as an essential socio-political consideration was contributed to by an understanding that soldiers fighting the Boer War were malnourished and that good nutrition was lacking in the working classes [31]. Modern European society faces issues of a similar magnitude. Without a strong policy focus to support good diet quality, prioritising lower income countries and lower education groups, large sections of European populations may have suboptimal intakes, with significant health and economic impacts.

This work is the first to harmonise and pool the individual-level survey data from national dietary surveys across WHO Member States, spanning all regions of Europe. This provides the largest representative dietary survey review of diet across WHO Europe, and evidence on which to base policy. The individual-level data harmonisation, with statistical analyses stratified by sex, and using European age standardisation based on the most recent ESP [17], facilitates between-country comparisons of nationally representative nutrient intakes to a degree not previously achieved.

The exploration of GDP and educational level in relation to nutrient intakes, is a previously under-studied area. In particular, this is the first time that individual participant data has been analysed in this way to explore the interactions between socioeconomic status, education and diet. Our analyses highlight the need for future research to further explore nutrient intakes in disadvantaged groups across Europe.

It is not possible to adequately evaluate these associations using summary reports alone, because not all surveys report on socioeconomic status or education [5]. We use education in part as a proxy for socioeconomic status. This has previously been used in relevant literature and official reports, including the Global Burden of Disease socio-demographic index [32] and the Euro-Peristat report [33]. However, although efforts at harmonising national dietary surveys, such as the EU MENU project, are progressing, this is limited to the EU [34]. In terms of harmonising educational level, although education was the best available indicator for individual socioeconomic status and broadly compatible education groups were created, it is possible that different countries expect different standards at each level.

The analyses have limitations. Firstly, the survey data used were cross-sectional, which does not demonstrate direction of association and therefore cannot show a causal relationship [35]. Socioeconomic inequalities were harder to assess at national level; with only 12 countries on which to test associations between nutrient intakes and GDP, the simple national level regression analysis lacked sufficient power to adequately test for significance or estimate associations with great precision. However, utilising the harmonised and pooled individual-level data improved our ability to detect associations. Nevertheless, there were few participants in the survey from Macedonia, resulting in wide confidence intervals and greater uncertainty in the lower education groups for total folate. In the Estonian survey, the lower education group contained fewer than three individuals, so associations for this group were not included to avoid statistical disclosure.

A further limitation is the nutrient composition databases from which nutrient intake values are derived. Not all countries’ databases are necessarily equally comprehensive or up-to-date; some mean nutrient intake values may therefore be less accurate. Differential treatment of fortification in national food composition databases may also make associations less reliable. Nutrient values may be calculated differently across countries, and some countries may not routinely analyse for certain nutrients. For example, TFA coverage in the Estonian database is poor and Sweden no longer report TFAs because the average population intake is below 1%E and therefore no longer a public health concern [36]. Nutrient values are based on a composite sample of a limited selection of foods. These may not include foods typically consumed by population subgroups, such as ethnic or street food. These subgroups may therefore have higher or lower intakes than the population average, hiding further potential health inequalities.

Despite harmonisation of data, the biggest limitation remains the heterogeneity in methods between the national dietary surveys. The surveys used different dietary assessment tools, so comparisons between countries must still be treated with caution. However, to reduce selection bias, we followed EFSA recommendations to include under-reporters of energy intake [18]. This removes another source of variation found in survey summary reports [4, 5]. Differential under-reporting means that true intakes are not necessarily reflected by the data. Under-reporting may be particularly affected by educational attainment [37], justifying our focus on macronutrient densities.

## Conclusion

This paper draws together individual datasets from national dietary surveys across 12 WHO European Member States. Potential socioeconomic inequalities were investigated by assessing selected nutrient intakes by GDP and education. These analyses can inform future research and policy development. National dietary survey data can enable exploration of variation between countries, as well as investigating nutrient intakes by demographic parameters and assessment of inequalities in disadvantaged groups. To aid this and facilitate valid data pooling, national dietary survey harmonisation should be encouraged and data made publicly accessible.

Inequalities between and within countries were shown; higher GDP countries had higher total sugar intakes. Within-country associations between lower education and higher intakes were particularly pronounced for intakes of the macronutrients studied, suggesting overall poorer diet quality. In countries with lower GDP having less education was associated with having a lower mean energy intake and higher education with having higher fat intakes. In contrast, higher education in higher GDP countries was associated with lower fat intakes. Lower education groups generally had lower micronutrient intakes. Education mitigated against the influence of GDP on nutrient intakes, suggesting that socioeconomic factors operate on national and individual levels. Having a higher education may mitigate against the increased fats intakes seen with increased GDP, and having lower education may weaken beneficial increases in total folate.

It is critical for countries to understand that increasing the educational level of their population will lead to better nourished populations, and the ability to improve GDP. Policies should therefore be put in place to achieve this.

## Supporting information

S1 Appendix National dietary survey datasets obtained. (DOCX)

S2 Appendix Age standardised mean adult energy and nutrient intakes in the WHO European Region by sex. (DOCX)

S3 Appendix Association between mean nutrient intakes and GDP in 12 countries of the WHO European Region. (DOCX)

S4 Appendix Age standardised mean energy and nutrient intakes for adult men in the WHO European Region by educational status. (DOCX)

S5 Appendix Age standardised mean energy and nutrient intakes for adult women in the WHO European Region by educational status. (DOCX)

S6 Appendix Association between nutrient intake and educational status, by sex, adjusted for age and GDP. (DOCX)
