# Supplementary material for: Inequalities in education and national income are associated with poorer diet: Pooled analysis of individual participant data across 12 European countries
Source: PLoS One. 2020 May 7;15(5):e0232447. doi: 10.1371/journal.pone.0232447 (PMC7205203; doi:10.1371/journal.pone.0232447)
Supplement: S1 Appendix — (DOCX) [file pone.0232447.s001.docx]

## **S1.Appendix – National Dietary Survey Datasets Obtained**

| **Country** | **Survey** | **Data Collection** | **Dietary Methodology** | **Total n*** | **Male** | | | **Female** | | |
| --- | --- | --- | --- | --- | --- | --- | --- | --- | --- | --- |
|  |  |  |  |  | **Lower education** | **Intermediate education** | **Higher education** | **Lower education** | **Intermediate education** | **Higher education** |
| Macedonia | First Macedonian Food Consumption Survey | 2015 | 2*24h recall | 387 | 6 | 123 | 40 | 16 | 136 | 66 |
| Kazakhstan | Nutritional and health status survey of the population in Kazakhstan | 2008 | 2*24h recall | 3071 | 17 | 1116 | 268 | 25 | 1270 | 375 |
| Hungary | Hungarian Diet & Nutritional Status Survey (OTÁP 2014) | 2014 | 3-day non-consecutive diary | 663 | 30 | 181 | 86 | 34 | 209 | 123 |
| Estonia | National Dietary Survey | 2014-2015 | 2*24h recall | 2573 | 2 | 518 | 215 | 2 | 988 | 848 |
| France | Individual National Food Consumption Survey (INCA2) | 2006-2007 | 7-day consecutive diary | 2235 | 25 | 589 | 302 | 47 | 839 | 433 |
| UK | National Diet and Nutrition Survey Rolling Programme Y7-8 (NDNS RP 2014-2016 ) | 2014-2016 | 4-day consecutive diary | 988 | 67 | 164 | 180 | 85 | 229 | 263 |
| Finland | The National FINDIET 2012 survey (FINRISK) | 2012 | 2*24h consecutive recall | 1283 | 187 | 191 | 200 | 235 | 218 | 252 |
| Sweden | Riksmaten 2010-2011 Swedish Adults Dietary Survey | 2010-2011 | 4-day consecutive web-based diary | 1405 | 72 | 267 | 272 | 53 | 334 | 407 |
| Germany | German National Nutrition Survey II (NVSII) | 2005-2007 | Diet history interview. | 10090 | 46 | 3129 | 1371 | 49 | 4106 | 1389 |
| Denmark | Danish National Survey of Diet and Physical Activity (DANSDA) | 2011-2013 | 7-day consecutive pre-coded diary | 2355 | 138 | 723 | 267 | 139 | 646 | 442 |
| Netherlands | Dutch National Food Consumption Survey 2007-2010 (DNFCS 2007-10) | 2007-2010 | 2*24h recall | 1933 | 282 | 460 | 222 | 338 | 432 | 199 |
| Austria | Austrian nutrition report 2012 (OSES) | 2010-2012 | 2*24h recall, non-consecutive diary | 351 | 56 | 42 | 47 | 76 | 70 | 60 |

* Unweighted numbers of adults aged 19-64y used in analyses.

NB – countries are ordered by GDP from lowest to highest.

## 
