# Supplementary material for: Inequalities in education and national income are associated with poorer diet: Pooled analysis of individual participant data across 12 European countries
Source: PLoS One. 2020 May 7;15(5):e0232447. doi: 10.1371/journal.pone.0232447 (PMC7205203; doi:10.1371/journal.pone.0232447)
Supplement: S2 Appendix — (DOCX) [file pone.0232447.s002.docx]

## **S2.Appendix – Age standardised mean adult energy and nutrient intakes in the WHO European Region by sex**

|  | **Male** | | | | **Female** | | | |
| --- | --- | --- | --- | --- | --- | --- | --- | --- |
| **Energy (kcal)** | N | Mean | 95% CI | | N | Mean | 95% CI | |
| Macedonia | 169 | 2705 | 2544 | 2866 | 218 | 2051 | 1930 | 2172 |
| Kazakhstan | 1401 | 2178 | 2138 | 2217 | 1670 | 1788 | 1756 | 1820 |
| Hungary | 297 | 2800 | 2676 | 2923 | 366 | 2099 | 1999 | 2198 |
| Estonia | 735 | 2157 | 2091 | 2224 | 1839 | 1535 | 1503 | 1566 |
| France | 919 | 2275 | 2227 | 2324 | 1321 | 1711 | 1679 | 1743 |
| UK | 450 | 2103 | 2030 | 2176 | 632 | 1628 | 1580 | 1675 |
| Finland | 585 | 2250 | 2175 | 2325 | 710 | 1730 | 1683 | 1777 |
| Sweden | 612 | 2269 | 2202 | 2335 | 794 | 1788 | 1746 | 1830 |
| Germany | 4665 | 2719 | 2681 | 2757 | 5666 | 2023 | 1995 | 2051 |
| Denmark | 1172 | 2690 | 2624 | 2755 | 1259 | 2000 | 1951 | 2048 |
| Netherlands | 964 | 2645 | 2594 | 2696 | 969 | 1960 | 1922 | 1997 |
| Austria | 147 | 2225 | 2125 | 2324 | 225 | 1868 | 1794 | 1942 |
| **Total Fat (%E)** | N | Mean | 95% CI | | N | Mean | 95% CI | |
| Macedonia | 169 | 33 | 31 | 35 | 218 | 32 | 31 | 33 |
| Kazakhstan | 1401 | 34 | 34 | 35 | 1670 | 35 | 35 | 36 |
| Hungary | 297 | 39 | 38 | 39 | 366 | 37 | 37 | 38 |
| Estonia | 735 | 36 | 35 | 36 | 1839 | 36 | 35 | 36 |
| France | 919 | 36 | 36 | 37 | 1321 | 39 | 38 | 39 |
| UK | 450 | 33 | 32 | 33 | 632 | 34 | 33 | 34 |
| Finland | 585 | 37 | 36 | 38 | 710 | 36 | 36 | 37 |
| Sweden | 612 | 35 | 34 | 35 | 794 | 35 | 35 | 36 |
| Germany | 4665 | 35 | 35 | 35 | 5666 | 34 | 33 | 34 |
| Denmark | 1172 | 37 | 36 | 37 | 1259 | 37 | 36 | 37 |
| Netherlands | 964 | 34 | 34 | 35 | 969 | 34 | 33 | 35 |
| Austria | 147 | 36 | 35 | 37 | 225 | 35 | 34 | 36 |
| **TFA (%E)** | N | Mean | 95% CI | | N | Mean | 95% CI | |
| Denmark | 1172 | 0·56 | 0·54 | 0·57 | 1259 | 0·57 | 0·55 | 0·58 |
| Macedonia | 169 | 0·50 | 0·41 | 0·59 | 218 | 0·49 | 0·42 | 0·56 |
| Kazakhstan | 1401 | 0·63 | 0·58 | 0·68 | 1670 | 0·61 | 0·56 | 0·65 |
| Estonia | 735 | 0·25 | 0·24 | 0·27 | 1839 | 0·27 | 0·26 | 0·28 |
| UK | 450 | 0·46 | 0·43 | 0·48 | 632 | 0·48 | 0·46 | 0·50 |
| Finland | 585 | 0·42 | 0·40 | 0·44 | 710 | 0·42 | 0·40 | 0·44 |
| Netherlands | 964 | 0·55 | 0·53 | 0·57 | 969 | 0·59 | 0·57 | 0·62 |
| **Total Sugar* (%E)** | N | Mean | 95% CI | | N | Mean | 95% CI | |
| Macedonia | 169 | 9 | 7 | 10 | 218 | 11 | 10 | 12 |
| Kazakhstan | 1401 | 19 | 18 | 19 | 1670 | 21 | 21 | 22 |
| Hungary | 297 | 16 | 15 | 17 | 366 | 19 | 18 | 20 |
| Estonia | 735 | 18 | 18 | 19 | 1839 | 22 | 22 | 23 |
| France | 919 | 16 | 16 | 17 | 1321 | 19 | 19 | 20 |
| UK | 450 | 18 | 17 | 19 | 632 | 20 | 19 | 21 |
| Finland | 585 | 20 | 19 | 21 | 710 | 21 | 21 | 22 |
| Sweden | 612 | 16 | 15 | 16 | 794 | 19 | 18 | 19 |
| Germany | 4665 | 22 | 22 | 22 | 5666 | 26 | 26 | 26 |
| Denmark | 1172 | 17 | 17 | 18 | 1259 | 18 | 18 | 19 |
| Netherlands | 964 | 20 | 19 | 20 | 969 | 21 | 21 | 22 |
| Austria | 147 | 18 | 17 | 19 | 225 | 20 | 19 | 21 |
| **Iron (mg)** | N | Mean | 95% CI | | N | Mean | 95% CI | |
| Macedonia | 169 | 14·8 | 13·8 | 15·9 | 218 | 11·7 | 10·9 | 12·5 |
| Kazakhstan | 1401 | 13·8 | 13·5 | 14·1 | 1670 | 11·6 | 11·3 | 12·0 |
| Hungary | 297 | 12·4 | 11·8 | 12·9 | 366 | 9·9 | 9·4 | 10·3 |
| Estonia | 735 | 13·8 | 13·1 | 14·5 | 1839 | 10·0 | 9·7 | 10·3 |
| France | 919 | 13·6 | 13·1 | 14·1 | 1321 | 10·7 | 10·4 | 11·0 |
| UK | 450 | 11·6 | 11·1 | 12·2 | 632 | 9·2 | 8·9 | 9·6 |
| Finland | 585 | 12·9 | 12·3 | 13·4 | 710 | 10·3 | 9·9 | 10·6 |
| Sweden | 612 | 11·5 | 11·2 | 11·9 | 794 | 9·5 | 9·2 | 9·8 |
| Germany | 4665 | 14·3 | 14·2 | 14·5 | 5666 | 11·5 | 11·4 | 11·6 |
| Denmark | 1172 | 12·8 | 12·5 | 13·1 | 1259 | 9·6 | 9·4 | 9·9 |
| Netherlands | 964 | 11·9 | 11·6 | 12·1 | 969 | 9·8 | 9·6 | 10·0 |
| Austria | 147 | 12·0 | 11·4 | 12·5 | 225 | 10·9 | 10·4 | 11·4 |
| **Total Folate (µg)** | N | Mean | 95% CI | | N | Mean | 95% CI | |
| Macedonia | 169 | 462 | 394 | 530 | 218 | 364 | 306 | 422 |
| Kazakhstan | 1401 | 124 | 121 | 128 | 1670 | 107 | 104 | 110 |
| Hungary | 297 | 170 | 158 | 181 | 366 | 143 | 133 | 152 |
| Estonia | 735 | 206 | 199 | 213 | 1839 | 162 | 157 | 166 |
| France | 919 | 274 | 266 | 282 | 1321 | 246 | 239 | 252 |
| UK | 450 | 264 | 251 | 277 | 632 | 214 | 204 | 224 |
| Finland | 585 | 283 | 262 | 305 | 710 | 230 | 222 | 237 |
| Sweden | 612 | 260 | 252 | 268 | 794 | 245 | 238 | 253 |
| Germany | 4665 | 349 | 337 | 361 | 5666 | 300 | 293 | 307 |
| Denmark | 1172 | 361 | 351 | 372 | 1259 | 314 | 304 | 323 |
| Netherlands | 964 | 303 | 295 | 312 | 969 | 248 | 240 | 255 |
| Austria | 147 | 211 | 198 | 224 | 225 | 211 | 199 | 223 |
| **Vitamin D (µg)** | N | Mean | 95% CI | | N | Mean | 95% CI | |
| Macedonia | 169 | 6·3 | 3·6 | 9·0 | 218 | 3·3 | 1·9 | 4·7 |
| Kazakhstan | 1401 | 1·1 | 1·0 | 1·2 | 1670 | 0·8 | 0·8 | 0·9 |
| Hungary | 297 | 2·7 | 2·4 | 2·9 | 366 | 2·1 | 1·9 | 2·2 |
| Estonia | 735 | 5·6 | 4·9 | 6·2 | 1839 | 4·1 | 3·7 | 4·4 |
| France | 919 | 2·5 | 2·4 | 2·6 | 1321 | 2·3 | 2·1 | 2·4 |
| UK | 450 | 2·8 | 2·6 | 3·1 | 632 | 2·5 | 2·3 | 2·7 |
| Finland | 585 | 10·7 | 9·9 | 11·4 | 710 | 8·2 | 7·8 | 8·7 |
| Sweden | 612 | 7·1 | 6·6 | 7·5 | 794 | 6·0 | 5·7 | 6·3 |
| Germany | 4665 | 3·5 | 3·4 | 3·6 | 5666 | 2·6 | 2·5 | 2·7 |
| Denmark | 1172 | 4·6 | 4·3 | 4·8 | 1259 | 3·7 | 3·5 | 4·0 |
| Netherlands | 964 | 4·0 | 3·9 | 4·2 | 969 | 3·1 | 2·9 | 3·3 |

* Where total sugar was not labelled within datasets as a single variable of that name, it was defined as monosaccharides plus disaccharides and a variable created to denote this value.

NB – countries are ordered by GDP from lowest to highest.
