# Supplementary material for: Inequalities in education and national income are associated with poorer diet: Pooled analysis of individual participant data across 12 European countries
Source: PLoS One. 2020 May 7;15(5):e0232447. doi: 10.1371/journal.pone.0232447 (PMC7205203; doi:10.1371/journal.pone.0232447)
Supplement: S3 Appendix — (DOCX) [file pone.0232447.s003.docx]

## **S3.Appendix – Association between mean nutrient intakes and GDP in 12 countries of the WHO European Region.**

|  | **Slope*** | **95% CI** | | **p-value** |
| --- | --- | --- | --- | --- |
| Energy (kcal) | -57 | -419 | 305 | 0·7 |
| Total fat (%E) | 1·0 | -1·8 | 3·8 | 0·4 |
| TFA (%E) | -0·006 | -0·291 | 0·279 | 1·0 |
| Total sugar (%E) | 5·0 | 0·6 | 9·3 | 0·03 |
| Iron (mg) | -1·28 | -2·58 | 0·02 | 0·05 |
| Total folate (µg) | 12 | -126 | 150 | 0·2 |
| Vitamin D (µg) | 1·4 | -2·7 | 5·5 | 0·5 |

*Slope represents the change in nutrient intake (per unit specified) for each 10% increase in GDP.
