# Supplementary material for: Inequalities in education and national income are associated with poorer diet: Pooled analysis of individual participant data across 12 European countries
Source: PLoS One. 2020 May 7;15(5):e0232447. doi: 10.1371/journal.pone.0232447 (PMC7205203; doi:10.1371/journal.pone.0232447)
Supplement: S4 Appendix — (DOCX) [file pone.0232447.s004.docx]

## **S4.Appendix – Age standardised mean energy and nutrient intakes for adult men in the WHO European Region by educational status**

|  | **Lower Education** | | | | **Intermediate Education** | | | | **Higher Education** | | | |
| --- | --- | --- | --- | --- | --- | --- | --- | --- | --- | --- | --- | --- |
| **Energy (kcal)** | N | Mean | 95% CI | | N | Mean | 95% CI | | N | Mean | 95% CI | |
| Macedonia | 6 | 2242 | 2168 | 2315 | 123 | 2593 | 2374 | 2813 | 40 | 2812 | 2492 | 3132 |
| Kazakhstan | 17 | 1843 | 1641 | 2045 | 1116 | 2147 | 2105 | 2188 | 268 | 2317 | 2213 | 2422 |
| Hungary | 30 | 2864 | 2634 | 3094 | 181 | 2877 | 2737 | 3016 | 86 | 2607 | 2464 | 2751 |
| Estonia* | 2 | N/A | N/A | N/A | 518 | 2129 | 2049 | 2208 | 215 | 2286 | 2188 | 2383 |
| France | 25 | 2103 | 1971 | 2235 | 589 | 2266 | 2207 | 2325 | 302 | 2337 | 2257 | 2417 |
| UK | 67 | 2007 | 1875 | 2139 | 164 | 2028 | 1939 | 2118 | 180 | 2168 | 2072 | 2265 |
| Finland | 187 | 2350 | 2193 | 2507 | 191 | 2202 | 2070 | 2333 | 200 | 2204 | 2104 | 2304 |
| Sweden | 72 | 2089 | 1945 | 2232 | 267 | 2249 | 2073 | 2426 | 272 | 2318 | 2245 | 2391 |
| Germany | 46 | 2378 | 2104 | 2653 | 3129 | 2687 | 2646 | 2729 | 1371 | 2805 | 2722 | 2888 |
| Denmark | 138 | 2781 | 2633 | 2929 | 723 | 2728 | 2644 | 2812 | 267 | 2632 | 2525 | 2740 |
| Netherlands | 282 | 2701 | 2605 | 2796 | 460 | 2675 | 2607 | 2744 | 222 | 2516 | 2430 | 2601 |
| Austria | 56 | 2206 | 2098 | 2315 | 42 | 2182 | 2028 | 2335 | 47 | 2217 | 2089 | 2346 |
| **Total Fat (%E)** | N | Mean | 95% CI | | N | Mean | 95% CI | | N | Mean | 95% CI | |
| Macedonia | 6 | 32 | 30 | 34 | 123 | 33 | 31 | 35 | 40 | 34 | 31 | 38 |
| Kazakhstan | 17 | 29 | 26 | 33 | 1116 | 34 | 33 | 34 | 268 | 37 | 36 | 39 |
| Hungary | 30 | 38 | 37 | 40 | 181 | 39 | 38 | 40 | 86 | 38 | 37 | 39 |
| Estonia* | 2 | N/A | N/A | N/A | 518 | 35 | 35 | 36 | 215 | 36 | 35 | 37 |
| France | 25 | 36 | 34 | 38 | 589 | 36 | 36 | 37 | 302 | 36 | 36 | 37 |
| UK | 67 | 32 | 30 | 33 | 164 | 32 | 31 | 33 | 180 | 33 | 32 | 34 |
| Finland | 187 | 37 | 36 | 39 | 191 | 35 | 33 | 37 | 200 | 37 | 36 | 39 |
| Sweden | 72 | 35 | 34 | 37 | 267 | 35 | 34 | 36 | 272 | 35 | 34 | 36 |
| Germany | 46 | 34 | 33 | 36 | 3129 | 35 | 35 | 35 | 1371 | 35 | 34 | 35 |
| Denmark | 138 | 37 | 36 | 38 | 723 | 37 | 37 | 38 | 267 | 36 | 35 | 37 |
| Netherlands | 282 | 35 | 34 | 36 | 460 | 34 | 34 | 35 | 222 | 34 | 33 | 34 |
| Austria | 56 | 35 | 34 | 36 | 42 | 37 | 36 | 39 | 47 | 37 | 36 | 39 |
| **TFA (%E)** | N | Mean | 95% CI | | N | Mean | 95% CI | | N | Mean | 95% CI | |
| Denmark | 138 | 0·56 | 0·53 | 0·60 | 723 | 0·56 | 0·54 | 0·58 | 267 | 0·54 | 0·51 | 0·57 |
| Macedonia | 6 | 0·34 | 0·20 | 0·48 | 123 | 0·53 | 0·43 | 0·63 | 40 | 0·54 | 0·36 | 0·72 |
| Kazakhstan | 17 | 0·41 | 0·24 | 0·58 | 1116 | 0·60 | 0·54 | 0·66 | 268 | 0·80 | 0·67 | 0·93 |
| Estonia* | 2 | N/A | N/A | N/A | 518 | 0·24 | 0·23 | 0·26 | 215 | 0·29 | 0·27 | 0·31 |
| UK | 67 | 0·44 | 0·41 | 0·48 | 164 | 0·45 | 0·41 | 0·48 | 180 | 0·47 | 0·44 | 0·50 |
| Finland | 187 | 0·43 | 0·41 | 0·46 | 191 | 0·42 | 0·38 | 0·45 | 200 | 0·41 | 0·38 | 0·43 |
| Netherlands | 282 | 0·56 | 0·52 | 0·60 | 460 | 0·55 | 0·52 | 0·58 | 222 | 0·55 | 0·51 | 0·58 |
| **Total Sugar (%E)**** | N | Mean | 95% CI | | N | Mean | 95% CI | | N | Mean | 95% CI | |
| Macedonia | 6 | 12 | 10 | 14 | 123 | 8 | 7 | 10 | 40 | 8 | 7 | 10 |
| Kazakhstan | 17 | 19 | 16 | 23 | 1116 | 19 | 18 | 19 | 268 | 18 | 17 | 19 |
| Hungary | 30 | 13 | 11 | 16 | 181 | 16 | 15 | 17 | 86 | 17 | 16 | 18 |
| Estonia* | 2 | N/A | N/A | N/A | 518 | 18 | 17 | 18 | 215 | 21 | 20 | 22 |
| France | 25 | 13 | 11 | 14 | 589 | 16 | 16 | 17 | 302 | 17 | 16 | 18 |
| UK | 67 | 20 | 19 | 21 | 164 | 18 | 17 | 19 | 180 | 18 | 17 | 19 |
| Finland | 187 | 19 | 17 | 20 | 191 | 21 | 19 | 23 | 200 | 20 | 18 | 21 |
| Sweden | 72 | 15 | 14 | 17 | 267 | 15 | 14 | 16 | 272 | 16 | 15 | 17 |
| Germany | 46 | 23 | 21 | 25 | 3129 | 21 | 21 | 22 | 1371 | 23 | 22 | 24 |
| Denmark | 138 | 18 | 16 | 19 | 723 | 17 | 16 | 17 | 267 | 17 | 16 | 18 |
| Netherlands | 282 | 20 | 19 | 21 | 460 | 20 | 19 | 21 | 222 | 20 | 19 | 21 |
| Austria | 56 | 18 | 17 | 19 | 42 | 20 | 18 | 22 | 47 | 18 | 17 | 19 |
| **Iron (mg)** | N | Mean | 95% CI | | N | Mean | 95% CI | | N | Mean | 95% CI | |
| Macedonia | 6 | 13·7 | 12·3 | 15·2 | 123 | 14·4 | 13·1 | 15·7 | 40 | 14·9 | 13·1 | 16·7 |
| Kazakhstan | 17 | 11·5 | 10·1 | 12·9 | 1116 | 13·6 | 13·2 | 13·9 | 268 | 15·0 | 14·2 | 15·9 |
| Hungary | 30 | 11·8 | 10·5 | 13·0 | 181 | 12·7 | 12·0 | 13·4 | 86 | 12·4 | 11·7 | 13·1 |
| Estonia* | 2 | N/A | N/A | N/A | 518 | 13·3 | 12·6 | 13·9 | 215 | 15·4 | 13·7 | 17·1 |
| France | 25 | 12·8 | 11·0 | 14·6 | 589 | 13·4 | 12·8 | 13·9 | 302 | 14·3 | 13·6 | 14·9 |
| UK | 67 | 9·9 | 8·9 | 10·9 | 164 | 11·1 | 10·5 | 11·6 | 180 | 12·5 | 11·7 | 13·3 |
| Finland | 187 | 13·2 | 12·0 | 14·3 | 191 | 12·8 | 11·8 | 13·8 | 200 | 12·5 | 11·8 | 13·3 |
| Sweden | 72 | 10·1 | 9·2 | 10·9 | 267 | 11·1 | 10·3 | 11·9 | 272 | 12·3 | 11·8 | 12·8 |
| Germany | 46 | 12·2 | 11·0 | 13·4 | 3129 | 14·4 | 14·1 | 14·6 | 1371 | 14·5 | 14·1 | 14·9 |
| Denmark | 138 | 12·9 | 12·3 | 13·5 | 723 | 13·0 | 12·6 | 13·4 | 267 | 12·8 | 12·1 | 13·5 |
| Netherlands | 282 | 11·7 | 11·2 | 12·1 | 460 | 11·8 | 11·5 | 12·2 | 222 | 12·2 | 11·7 | 12·6 |
| Austria | 56 | 11·6 | 11·0 | 12·2 | 42 | 11·2 | 10·3 | 12·2 | 47 | 11·1 | 10·4 | 11·8 |
| **Total Folate (µg)** | N | Mean | 95% CI | | N | Mean | 95% CI | | N | Mean | 95% CI | |
| Macedonia | 6 | 793 | 656 | 930 | 123 | 382 | 348 | 415 | 40 | 447 | 365 | 529 |
| Kazakhstan | 17 | 104 | 91 | 117 | 1116 | 123 | 120 | 127 | 268 | 128 | 121 | 135 |
| Hungary | 30 | 131 | 116 | 146 | 181 | 176 | 160 | 191 | 86 | 186 | 171 | 202 |
| Estonia* | 2 | N/A | N/A | N/A | 518 | 197 | 189 | 205 | 215 | 233 | 223 | 244 |
| France | 25 | 242 | 204 | 279 | 589 | 271 | 262 | 280 | 302 | 287 | 274 | 299 |
| UK | 67 | 224 | 198 | 249 | 164 | 244 | 231 | 257 | 180 | 292 | 269 | 315 |
| Finland | 187 | 299 | 244 | 353 | 191 | 278 | 247 | 309 | 200 | 269 | 251 | 287 |
| Sweden | 72 | 230 | 214 | 246 | 267 | 245 | 229 | 261 | 272 | 280 | 268 | 293 |
| Germany | 46 | 274 | 240 | 308 | 3129 | 360 | 343 | 377 | 1371 | 331 | 313 | 349 |
| Denmark | 138 | 359 | 335 | 382 | 723 | 369 | 355 | 383 | 267 | 379 | 349 | 408 |
| Netherlands | 282 | 297 | 281 | 313 | 460 | 305 | 292 | 318 | 222 | 308 | 292 | 323 |
| Austria | 56 | 200 | 188 | 213 | 42 | 220 | 197 | 242 | 47 | 216 | 199 | 233 |
| **Vitamin D (µg)** | N | Mean | 95% CI | | N | Mean | 95% CI | | N | Mean | 95% CI | |
| Macedonia | 6 | 1·3 | 0·8 | 1·8 | 123 | 4·7 | 2·6 | 6·8 | 40 | 6·6 | 3·0 | 10·2 |
| Kazakhstan | 17 | 1·3 | 0·5 | 2·1 | 1116 | 1·1 | 1·0 | 1·2 | 268 | 1·4 | 1·1 | 1·7 |
| Hungary | 30 | 2·2 | 1·8 | 2·6 | 181 | 2·6 | 2·4 | 2·9 | 86 | 2·9 | 2·4 | 3·4 |
| Estonia* | 2 | N/A | N/A | N/A | 518 | 5·9 | 5·0 | 6·8 | 215 | 4·8 | 4·1 | 5·5 |
| France | 25 | 2·9 | 1·8 | 4·1 | 589 | 2·4 | 2·3 | 2·6 | 302 | 2·6 | 2·4 | 2·8 |
| UK | 67 | 2·8 | 2·3 | 3·2 | 164 | 2·8 | 2·5 | 3·1 | 180 | 2·9 | 2·5 | 3·4 |
| Finland | 187 | 12·0 | 10·6 | 13·5 | 191 | 10·6 | 9·2 | 12·1 | 200 | 9·9 | 9·0 | 10·9 |
| Sweden | 72 | 6·5 | 5·5 | 7·6 | 267 | 6·5 | 5·8 | 7·1 | 272 | 7·7 | 7·0 | 8·4 |
| Germany | 46 | 3·0 | 2·4 | 3·6 | 3129 | 3·6 | 3·5 | 3·7 | 1371 | 3·5 | 3·3 | 3·7 |
| Denmark | 138 | 3·9 | 3·4 | 4·4 | 723 | 4·9 | 4·6 | 5·3 | 267 | 5·0 | 4·3 | 5·6 |
| Netherlands | 282 | 4·2 | 3·9 | 4·5 | 460 | 4·1 | 3·8 | 4·3 | 222 | 3·5 | 3·2 | 3·7 |

*Lower educated Estonian male and female intakes not included due to n<3 individuals.

** Where total sugar was not labelled within datasets as a single variable of that name, it was defined as monosaccharides plus disaccharides and a variable created to denote this value.

NB – countries are ordered by GDP from lowest to highest.
