# Supplementary material for: Inequalities in education and national income are associated with poorer diet: Pooled analysis of individual participant data across 12 European countries
Source: PLoS One. 2020 May 7;15(5):e0232447. doi: 10.1371/journal.pone.0232447 (PMC7205203; doi:10.1371/journal.pone.0232447)
Supplement: S5 Appendix — (DOCX) [file pone.0232447.s005.docx]

## **S5.Appendix – Age standardised mean energy and nutrient intakes for adult women in the WHO European Region by educational status**

|  | **Lower Education** | | | | **Intermediate Education** | | | | **Higher Education** | | | |
| --- | --- | --- | --- | --- | --- | --- | --- | --- | --- | --- | --- | --- |
| **Energy (kcal)** | N | Mean | 95% CI | | N | Mean | 95% CI | | N | Mean | 95% CI | |
| Macedonia | 16 | 1697 | 1451 | 1944 | 136 | 2059 | 1908 | 2211 | 66 | 2129 | 1961 | 2297 |
| Kazakhstan | 25 | 1678 | 1427 | 1930 | 1270 | 1783 | 1748 | 1819 | 375 | 1793 | 1686 | 1900 |
| Hungary | 34 | 2237 | 2017 | 2457 | 209 | 2015 | 1917 | 2113 | 123 | 2008 | 1913 | 2104 |
| Estonia* | 2 | N/A | N/A | N/A | 988 | 1473 | 1429 | 1516 | 848 | 1626 | 1585 | 1666 |
| France | 47 | 1586 | 1470 | 1703 | 839 | 1684 | 1643 | 1724 | 433 | 1788 | 1741 | 1835 |
| UK | 85 | 1420 | 1302 | 1538 | 229 | 1595 | 1514 | 1675 | 263 | 1682 | 1609 | 1754 |
| Finland | 235 | 1732 | 1640 | 1824 | 218 | 1690 | 1622 | 1758 | 252 | 1755 | 1679 | 1831 |
| Sweden | 53 | 1349 | 1242 | 1456 | 334 | 1775 | 1699 | 1850 | 407 | 1858 | 1809 | 1906 |
| Germany | 49 | 2127 | 1970 | 2284 | 4106 | 2011 | 1986 | 2037 | 1389 | 2065 | 1983 | 2147 |
| Denmark | 139 | 1877 | 1779 | 1974 | 646 | 2006 | 1945 | 2067 | 442 | 2032 | 1956 | 2107 |
| Netherlands | 338 | 1977 | 1911 | 2043 | 432 | 2004 | 1944 | 2064 | 199 | 1874 | 1806 | 1943 |
| Austria | 76 | 1753 | 1651 | 1855 | 70 | 1881 | 1760 | 2001 | 60 | 2002 | 1866 | 2139 |
| **Total Fat (%E)** | N | Mean | 95% CI | | N | Mean | 95% CI | | N | Mean | 95% CI | |
| Macedonia | 16 | 29 | 27 | 32 | 136 | 31 | 30 | 33 | 66 | 34 | 32 | 37 |
| Kazakhstan | 25 | 29 | 27 | 31 | 1270 | 35 | 34 | 35 | 375 | 36 | 34 | 37 |
| Hungary | 34 | 36 | 35 | 37 | 209 | 37 | 37 | 38 | 123 | 38 | 37 | 39 |
| Estonia* | 2 | N/A | N/A | N/A | 988 | 36 | 35 | 36 | 848 | 36 | 35 | 36 |
| France | 47 | 38 | 36 | 39 | 839 | 38 | 38 | 39 | 433 | 39 | 38 | 39 |
| UK | 85 | 33 | 32 | 35 | 229 | 33 | 32 | 34 | 263 | 35 | 34 | 36 |
| Finland | 235 | 37 | 35 | 38 | 218 | 36 | 34 | 37 | 252 | 36 | 35 | 37 |
| Sweden | 53 | 33 | 32 | 35 | 334 | 36 | 35 | 36 | 407 | 35 | 35 | 36 |
| Germany | 49 | 37 | 35 | 39 | 4106 | 34 | 33 | 34 | 1389 | 34 | 33 | 34 |
| Denmark | 139 | 37 | 36 | 39 | 646 | 37 | 36 | 37 | 442 | 36 | 36 | 37 |
| Netherlands | 338 | 35 | 34 | 36 | 432 | 34 | 33 | 34 | 199 | 34 | 33 | 34 |
| Austria | 76 | 36 | 35 | 37 | 70 | 35 | 33 | 36 | 60 | 35 | 34 | 37 |
| **TFA (%E)** | N | Mean | 95% CI | | N | Mean | 95% CI | | N | Mean | 95% CI | |
| Denmark | 139 | 0·66 | 0·61 | 0·71 | 646 | 0·55 | 0·53 | 0·57 | 442 | 0·53 | 0·51 | 0·55 |
| Macedonia | 16 | 0·40 | 0·21 | 0·60 | 136 | 0·46 | 0·38 | 0·54 | 66 | 0·52 | 0·39 | 0·65 |
| Kazakhstan | 25 | 0·31 | 0·14 | 0·47 | 1270 | 0·60 | 0·54 | 0·65 | 375 | 0·60 | 0·52 | 0·69 |
| Estonia* | 2 | N/A | N/A | N/A | 988 | 0·26 | 0·25 | 0·27 | 848 | 0·29 | 0·27 | 0·31 |
| UK | 85 | 0·49 | 0·45 | 0·53 | 229 | 0·47 | 0·44 | 0·51 | 263 | 0·49 | 0·46 | 0·52 |
| Finland | 235 | 0·43 | 0·40 | 0·46 | 218 | 0·41 | 0·38 | 0·44 | 252 | 0·42 | 0·39 | 0·45 |
| Netherlands | 338 | 0·60 | 0·56 | 0·64 | 432 | 0·59 | 0·55 | 0·62 | 199 | 0·56 | 0·52 | 0·61 |
| **Total Sugar (%E)**** | N | Mean | 95% CI | | N | Mean | 95% CI | | N | Mean | 95% CI | |
| Macedonia | 16 | 11 | 9 | 13 | 136 | 10 | 9 | 11 | 66 | 13 | 11 | 15 |
| Kazakhstan | 25 | 25 | 22 | 28 | 1270 | 21 | 21 | 22 | 375 | 22 | 20 | 23 |
| Hungary | 34 | 15 | 13 | 16 | 209 | 19 | 18 | 20 | 123 | 20 | 19 | 21 |
| Estonia* | 2 | N/A | N/A | N/A | 988 | 21 | 21 | 22 | 848 | 23 | 22 | 24 |
| France | 47 | 18 | 15 | 20 | 839 | 19 | 19 | 20 | 433 | 20 | 19 | 20 |
| UK | 85 | 20 | 18 | 21 | 229 | 20 | 19 | 22 | 263 | 20 | 19 | 21 |
| Finland | 235 | 21 | 20 | 22 | 218 | 21 | 20 | 22 | 252 | 22 | 21 | 23 |
| Sweden | 53 | 17 | 16 | 19 | 334 | 19 | 18 | 20 | 407 | 18 | 18 | 19 |
| Germany | 49 | 25 | 23 | 27 | 4106 | 26 | 25 | 26 | 1389 | 27 | 26 | 27 |
| Denmark | 139 | 18 | 17 | 19 | 646 | 19 | 18 | 19 | 442 | 18 | 18 | 19 |
| Netherlands | 338 | 22 | 21 | 22 | 432 | 22 | 21 | 22 | 199 | 21 | 20 | 22 |
| Austria | 76 | 20 | 19 | 22 | 70 | 21 | 19 | 23 | 60 | 21 | 20 | 22 |
| **Iron (mg)** | N | Mean | 95% CI | | N | Mean | 95% CI | | N | Mean | 95% CI | |
| Macedonia | 16 | 11·3 | 9·9 | 12·8 | 136 | 11·8 | 10·9 | 12·7 | 66 | 11·4 | 10·2 | 12·6 |
| Kazakhstan | 25 | 9·3 | 7·7 | 10·9 | 1270 | 11·5 | 11·1 | 11·9 | 375 | 12·4 | 11·7 | 13·2 |
| Hungary | 34 | 9·5 | 8·7 | 10·4 | 209 | 9·6 | 9·1 | 10·1 | 123 | 10·0 | 9·4 | 10·6 |
| Estonia* | 2 | N/A | N/A | N/A | 988 | 9·5 | 9·2 | 9·9 | 848 | 10·8 | 10·4 | 11·3 |
| France | 47 | 9·9 | 9·2 | 10·6 | 839 | 10·5 | 10·1 | 10·8 | 433 | 11·4 | 10·9 | 11·9 |
| UK | 85 | 8·3 | 7·3 | 9·3 | 229 | 8·7 | 8·3 | 9·1 | 263 | 9·9 | 9·4 | 10·3 |
| Finland | 235 | 9·8 | 9·2 | 10·4 | 218 | 10·5 | 9·9 | 11·1 | 252 | 10·4 | 9·9 | 10·9 |
| Sweden | 53 | 8·1 | 7·5 | 8·7 | 334 | 9·0 | 8·5 | 9·5 | 407 | 10·2 | 9·8 | 10·5 |
| Germany | 49 | 11·3 | 10·5 | 12·1 | 4106 | 11·6 | 11·4 | 11·7 | 1389 | 11·5 | 11·1 | 11·9 |
| Denmark | 139 | 8·4 | 7·9 | 8·8 | 646 | 9·8 | 9·5 | 10·1 | 442 | 10·0 | 9·6 | 10·4 |
| Netherlands | 338 | 9·6 | 9·2 | 10·0 | 432 | 10·0 | 9·6 | 10·3 | 199 | 10·4 | 10·0 | 10·8 |
| Austria | 76 | 10·3 | 9·6 | 11·0 | 70 | 11·0 | 10·0 | 11·9 | 60 | 11·3 | 10·5 | 12·1 |
| **Total Folate (µg)** | N | Mean | 95% CI | | N | Mean | 95% CI | | N | Mean | 95% CI | |
| Macedonia | 16 | 337 | 267 | 406 | 136 | 354 | 281 | 428 | 66 | 359 | 318 | 400 |
| Kazakhstan | 25 | 109 | 88 | 130 | 1270 | 107 | 104 | 110 | 375 | 101 | 96 | 106 |
| Hungary | 34 | 124 | 112 | 136 | 209 | 134 | 123 | 145 | 123 | 162 | 146 | 178 |
| Estonia* | 2 | N/A | N/A | N/A | 988 | 152 | 146 | 157 | 848 | 181 | 174 | 187 |
| France | 47 | 210 | 196 | 224 | 839 | 237 | 229 | 245 | 433 | 269 | 258 | 280 |
| UK | 85 | 191 | 164 | 218 | 229 | 194 | 183 | 204 | 263 | 229 | 217 | 242 |
| Finland | 235 | 212 | 199 | 225 | 218 | 238 | 224 | 253 | 252 | 237 | 225 | 249 |
| Sweden | 53 | 203 | 182 | 224 | 334 | 226 | 216 | 236 | 407 | 269 | 258 | 280 |
| Germany | 49 | 328 | 292 | 364 | 4106 | 302 | 294 | 310 | 1389 | 303 | 276 | 331 |
| Denmark | 139 | 265 | 246 | 283 | 646 | 324 | 308 | 339 | 442 | 335 | 320 | 351 |
| Netherlands | 338 | 234 | 220 | 247 | 432 | 251 | 240 | 262 | 199 | 261 | 247 | 276 |
| Austria | 76 | 204 | 188 | 220 | 70 | 236 | 197 | 275 | 60 | 234 | 218 | 251 |
| **Vitamin D (µg)** | N | Mean | 95% CI | | N | Mean | 95% CI | | N | Mean | 95% CI | |
| Macedonia | 16 | 1·1 | 0·8 | 1·4 | 136 | 3·3 | 1·8 | 4·8 | 66 | 3·8 | 1·8 | 5·9 |
| Kazakhstan | 25 | 0·7 | 0·4 | 0·9 | 1270 | 0·8 | 0·7 | 0·9 | 375 | 0·8 | 0·7 | 0·9 |
| Hungary | 34 | 2·3 | 1·9 | 2·6 | 209 | 2·0 | 1·9 | 2·2 | 123 | 2·0 | 1·8 | 2·2 |
| Estonia* | 2 | N/A | N/A | N/A | 988 | 4·0 | 3·6 | 4·4 | 848 | 4·4 | 3·8 | 5·0 |
| France | 47 | 1·9 | 1·6 | 2·2 | 839 | 2·2 | 2·0 | 2·3 | 433 | 2·4 | 2·2 | 2·6 |
| UK | 85 | 2·4 | 2·1 | 2·7 | 229 | 2·4 | 2·2 | 2·7 | 263 | 2·6 | 2·3 | 2·8 |
| Finland | 235 | 7·9 | 7·1 | 8·7 | 218 | 8·3 | 7·6 | 9·1 | 252 | 8·3 | 7·5 | 9·0 |
| Sweden | 53 | 4·7 | 3·8 | 5·5 | 334 | 5·8 | 5·3 | 6·3 | 407 | 6·5 | 6·1 | 7·0 |
| Germany | 49 | 2·7 | 1·9 | 3·5 | 4106 | 2·6 | 2·5 | 2·7 | 1389 | 2·5 | 2·4 | 2·7 |
| Netherlands | 338 | 3·1 | 2·9 | 3·3 | 432 | 3·3 | 3·0 | 3·5 | 199 | 2·9 | 2·5 | 3·3 |
| Denmark | 139 | 3·1 | 2·7 | 3·5 | 646 | 3·5 | 3·3 | 3·8 | 442 | 4·0 | 3·6 | 4·5 |

*Lower educated Estonian male and female intakes not included due to n<3 individuals.

** Where total sugar was not labelled within datasets as a single variable of that name, it was defined as monosaccharides plus disaccharides and a variable created to denote this value.

NB – countries are ordered by GDP from lowest to highest.
