# Supplementary material for: Inequalities in education and national income are associated with poorer diet: Pooled analysis of individual participant data across 12 European countries
Source: PLoS One. 2020 May 7;15(5):e0232447. doi: 10.1371/journal.pone.0232447 (PMC7205203; doi:10.1371/journal.pone.0232447)
Supplement: S6 Appendix — (DOCX) [file pone.0232447.s006.docx]

## **S6.Appendix – Association between nutrient intake and educational status, by sex, adjusted for age and GDP**

|  | **MEN – Lower vs. Intermediate** | | | **MEN – Higher vs. Intermediate** | | | **WOMEN – Lower vs. Intermediate** | | | **WOMEN – Higher vs. Intermediate** | | | **P-value**** |
| --- | --- | --- | --- | --- | --- | --- | --- | --- | --- | --- | --- | --- | --- |
|  | Slope* | 95% CI | | Slope* | 95% CI | | Slope* | 95% CI | | Slope* | 95% CI | |  |
| Energy (kcal) | -6 | -81 | 69 | 43 | -1 | 87 | -67 | -115 | -20 | -12 | -37 | 12 | 0·06 |
| Total fat (%E) | 0·9 | 0·3 | 1·5 | 0·2 | -0·2 | 0·5 | 0·9 | 0·3 | 1·4 | 0·4 | 0·1 | 0·7 | 0·7 |
| TFA (%E) | 0·07 | 0·04 | 0·10 | 0·01 | -0·02 | 0·04 | 0·07 | 0·04 | 0·11 | -0·01 | -0·04 | 0·02 | 0·5 |
| Total sugar (%E) | -1·1 | -1·8 | -0·5 | 0·9 | 0·5 | 1·2 | -3·0 | -3·5 | -2·4 | -0·2 | -0·6 | 0·1 | <0·001 |
| Iron (mg) | -1·4 | -1·7 | -1·0 | 0·4 | 0·1 | 0·7 | -1·3 | -1·6 | -1·0 | 0·1 | -0·1 | 0·2 | 0·1 |
| Total folate (µg) | -15 | -35 | 5 | -2 | -11 | 8 | -31 | -39 | -22 | 2 | -4 | 9 | 0·2 |
| Vitamin D (µg) | 1·4 | 1·0 | 1·9 | 0·2 | -0·01 | 0·4 | 0·8 | 0·6 | 1·1 | 0·5 | 0·4 | 0·7 | 0·004 |

*Slope represents the change in nutrient intake (per unit specified) for each 10% increase in GDP.

** P-value for difference between men and women in the association between education and nutrient intake, formally tested by the inclusion of the interaction between education group and sex.
